# Challenges in the dose calculation from urine measurements in routine internal monitoring of ^131^I and other radionuclides

**DOI:** 10.1007/s00411-025-01129-z

**Published:** 2025-05-27

**Authors:** Oliver Meisenberg, Ayesha Mohsin

**Affiliations:** 1 Federal Office for Radiation Protection (BfS), Medical and Occupational Radiation Protection, 85764 Oberschleißheim, Germany; 2 Pakistan Nuclear Regulatory Authority (PNRA), Environmental Monitoring and Dosimetry Labs, Islamabad, Pakistan

**Keywords:** Internal monitoring, Internal dosimetry, Radioiodine therapy, Occupational intakes of radionuclides, Standardisation

## Abstract

The measurement of 24-hour urine samples is one of the methods of routine monitoring of intakes of radionuclides. It is briefly mentioned in relevant documents by the International Commission on Radiological Protection that for ^131^I the strong decrease of the excretion within the first days after an intake makes the dose calculation from urine measurements unreliable when the time pattern of the intake is unknown. This can result in a major overestimation of the committed effective dose. For quantifying the influence of the time pattern of an intake on the dose, the results of the dose calculation for an acute intake at the midpoint of a monitoring interval (standard assumption) were compared with those for a chronic intake with varying daily activity. For ^131^I, aerosols type F, the standard assumption of an acute intake can lead to an overestimation of the calculated dose by a factor of 140 on average as compared to a chronic intake. Among other investigated radionuclides, the strongest overestimation was found for ^14^C, gas/vapour type F, when measured every 180 days (factor of 330), although this method complies with current criteria from the international standard ISO 20553. It is recommended that ISO 20553 is supplemented with a criterion that describes the reliability of a monitoring method under different time patterns of an intake additional to the existing criteria. This criterion should set an upper limit for the ratio of the dose calculations under the described assumptions.

## Introduction

**Fig. 1:**
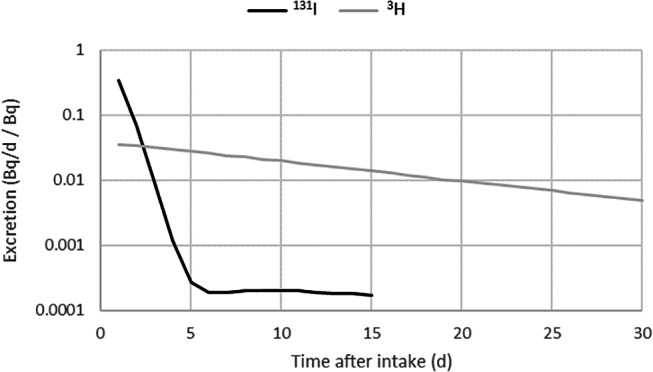
Excretion rates (for urine) of ^131^I (aerosols type F) with a strong decrease during the first days after an acute intake; for comparison, excretion rates of ^3^H (gas or vapour type V for very fast absorption into blood) exhibit a smooth curve. Both curves are drawn for the length of the respective monitoring interval. Values taken from ICRP 2022

^131^I is applied worldwide for the treatment of thyroid diseases. It is administered in the chemical form of sodium iodide (NaI) orally as capsules or liquid solutions, or intravenously in liquid form (Campennì et al. 2023). In particular, the liquid form results in a significant risk of intakes at workplaces where the radiopharmaceuticals are produced or administered. After an intake via inhalation or ingestion, the ^131^I accumulates in the thyroid due to its biokinetics. Most of the ^131^I that has been taken up into the thyroid will decay there instead of being released back into the blood. Nevertheless, small activities are also excreted in urine. Because of these particular biokinetics with a depot in the thyroid, the excretion via urine features a rapid decrease over more than 3 orders of magnitude during the first ca. 4 days and is approximately constant ca. from day 5 to 15 after an intake (Fig. 1).

Because of its emission of gamma radiation, internal monitoring of ^131^I can be conducted by means of thyroid counting (direct measurement of the activity in the thyroid) and by gamma spectrometry of 24-hour urine samples (in-vitro bioassay). However, several international organisations such as the International Atomic Energy Agency (IAEA) and the International Commission on Radiological Protection (ICRP) recommend to prefer direct measurements of ^131^I in the thyroid for a more reliable dose calculation when the time of intake is unknown (IAEA 1999; ICRP 2017). ICRP Publication 54 contains a warning against the monitoring of ^131^I by means of urine measurements unless an intake can be excluded during the last five days before the urine sampling (ICRP 1989). Despite these caveats, urine measurements are occasionally conducted for routine internal monitoring for ^131^I in a variety of regions (Díaz-Londoño et al. 2017; Happel et al. 2017; Jahan et al. 2024; Jeong et al. 2011; Lucena et al. 2007; Shoji et al. 2005). It is acknowledged that the measurement of ^131^I in urine has organisational advantages over a direct measurement: the possibility to send urine samples to a central internal monitoring service and the shared use of gamma-spectrometry systems that are available anyway. In some regions, urine measurements represent the only available method for the monitoring of ^131^I because of its simplicity and low costs.

How often direct measurements must be conducted or bioassay samples must be collected in routine internal monitoring (the monitoring interval) is determined by two criteria from ISO 20553, Eqs. 1–4: the factor-3 criterion and the 1-mSv criterion (ISO 2025). The former of these criteria depends only on the biokinetic data of the radionuclide whereas the latter is influenced by the detection limit of the monitoring method. For ^131^I, inhalation, type F, the monitoring interval for direct measurements and for urine bioassay is 15 days with achievable detection limits of 1 Bq in the thyroid and of 0.42 Bq/d in urine (ICRP 2017; calculated with a standard excretion rate of 1.4 L/d, which is the arithmetic mean of the standard excretion rates for males, 1.6 L/d, and females, 1.2 L/d, as also applied e.g. in ISO 20553; ICRP 2002). For compliance with the 1-mSv criterion, the detection limit in urine must be 0.63 Bq/d or smaller.

In contrast to special monitoring after incidents with a risk of intake at the workplace, the time of intake is typically not known in routine monitoring. Information about the most likely time of the intake can be gained from repeated measurements. However, this is laborious and not possible in all intake cases since it requires among others the absence of further intakes after the first measurement or urine collection. In some cases, the time of intake can also be determined from a comparison of the results of direct measurements and in-vitro bioassay, however this requires the availability of both methods.

It will be shown in the following that the dose calculation in routine internal monitoring of ^131^I can be severely unreliable when based on urine measurements leading to an overestimation of the dose. In extreme cases, this overestimation can be by more than two orders of magnitude unless the time of the intake is known. To substantiate the general warnings against the application of monitoring in urine, the associated unreliability of dose estimates will be quantified below under different assumptions regarding acute and chronic intake. For comparison, the doses will also be calculated based on monitoring of ^131^I by thyroid measurements. Besides, several radionuclides were selected as further examples, among them those particularly relevant for radiological protection (e.g. ^3^H, ^14^C, ^137^Cs, ^238^U) and radionuclides that feature a likewise strong decrease of the excretion rate within the first days after an intake (^14^C in the form of barium carbonate, ^35^S, ^238^U as type F). Even though several of these radionuclides can be measured in individuals only in urine (beside the universal method of workplace monitoring, i.e., measuring radioactivity concentrations in indoor air), the results indicate for which radionuclides it is highly relevant to distinguish between a possible chronic, repeated or acute intake. As a guidance for internal monitoring services, an additional criterion for the amendment of ISO 20553 will be proposed.

## Methodology

In routine internal monitoring, the standard assumption for dose calculations is an acute intake at the midpoint of the monitoring interval (i.e. one single intake per monitoring interval). This is meant to guarantee a balancing between overestimation and underestimation of the true effective dose over the course of several monitoring intervals in which one intake per interval occurs at different times. Due to the factor-3 criterion, each single dose cannot be underestimated more strongly than by 1/3; however, no criterion exists for limiting the overestimation of a dose.

Dose calculations for ^131^I measured in urine and in the thyroid and for various other nuclides were conducted under the following assumptions:

### Acute intake at the midpoint of each monitoring interval (standard assumption)

The effective dose *E* was calculated using the standard equation (Eq. 1):

1 $$\:E\left(t\right)=A/m\left(t\right)\cdot\:e$$

where *A* is the measured activity in the whole-body, organ or 24-hour urine sample, and *m*(*t*) is the value of the reference biokinetic function (retention or excretion), both resulting in the intake $\:I=A/m\left(t\right)$, *e* is the dose coefficient for the committed effective dose (also written as *e*(50) with the dose-commitment period of 50 years), and *t* = Δ*T*/2 is for an intake at the midpoint of the monitoring interval of Δ*T*.

### Acute intake at a single random day within each monitoring interval (with uniform probability for each day)

The average effective dose per monitoring interval equals the arithmetic mean of the effective doses calculated for each single day from 1 to the length of the monitoring interval Δ*T* in days (Eq. 2):

2 $$\:E=\sum\:_{i=1}^{{\Delta\:}T}E\left(t\right)/{\Delta\:}T$$

### Chronic intake with equal intake on every day of the monitoring interval

The effective dose *E* for one monitoring interval was calculated as follows: An arbitrary value *I*_starting_ of the daily intake (in Bq/d) was assumed as a starting point. With this value, the resulting measured activity *A*_starting_ in the whole body, organ or urine sample was calculated (Eq. 3):

3 $$\:{A}_{\text{starting}}=\sum\:_{t=1}^{{\Delta\:}T}{I}_{\text{starting}}\cdot\:m\left(t\right)$$

*A*_starting_ was compared with the measured activity *A* in the whole body, organ or urine sample for which the effective dose should be calculated and the daily intake *I* was scaled accordingly (Eq. 4):

4 $$\:I={I}_{\text{starting}}\cdot\:A/{A}_{\text{starting}}$$

The effective dose *E* was calculated from the sum of the daily intakes over the monitoring interval of length Δ*T* in days (Eq. 5):

5 $$\:E=I\cdot\:e\cdot\:{\Delta\:}T$$

This can also be expressed with the value of the biokinetic function for chronic intake (Eqs. 6, 7):

6 $$\:{m}_{\text{chronic}}\left({\Delta\:}T\right)\::=\sum\:_{t=1}^{{\Delta\:}T}m\left(t\right)$$

7 $$\:E=A/{m}_{\text{chronic}}\left({\Delta\:}T\right)\cdot\:e\cdot\:{\Delta\:}T$$

The value of the biokinetic function for chronic intake $\:{m}_{\text{chronic}}\left(t\right)$ (in units of Bq per Bq/d for the retention and Bq/d per Bq/d for the excretion) can be understood as the activity in the body or the daily excretion when a constant daily intake of 1 Bq occurred starting *t* days before.

Assumptions b) and c) have in common that intakes can occur on each day of an interval with uniform probability. The difference is that for assumption c) the intake has a constant activity for each day whereas for assumption b) the intake depends on its day within the interval in such a way that it yields the measured activity (higher intakes for earlier intakes, smaller intakes for later ones).

### Chronic intake with random intake on every day of each monitoring interval (uniform probability density for the intake)

The effective dose *E* for one monitoring interval was calculated similar to assumption c) with the difference that a set of random values of the daily intake, *I*_starting, *i*_ (*i* = 1…Δ*T*) was assumed as the starting point. 100,000 runs with different sets of random numbers were conducted for each radionuclide, resulting in distributions of possible effective doses under different time patterns of a chronic intake.

Random numbers were sampled from an exponential distribution from 0 to infinity:

8 $${I_{starting,\,i}} = - {I_{mean}} \cdot \ln \left( X \right)$$

where *I*_mean_ is the arithmetic mean of the distribution, equalling the activity that was determined for the constant chronic intake (assumption c)), and *X* is a set of random numbers uniformly distributed between 0 and 1.

In contrast to a uniform distribution, random numbers from an exponential distribution comply approximately with the Newcomb-Benford law. The Newcomb-Benford law describes an empirical statistical property of the values of many observations in nature (Benford 1938; Engel and Leuenberger 2003) so that it appeared reasonable to sample random values of the daily intake from a distribution that satisfies this law. In contrast to a log-uniform distribution, which complies with the Newcomb-Benford law exactly, the exponential distribution does not require the arbitrary choice of a lower and upper bound because of its finite integral from 0 to infinity; the arithmetic mean is the only parameter of the distribution as described in Eq. 8; this parameter was selected equalling the intake from assumption c). In contrast to a log-normal distribution, which also complies approximately with the Newcomb-Benford law depending on the choice of its parameters, the exponential distribution emphasises small values, making it a reasonable distribution for including also very small or negligible daily intakes.

All dose calculations were conducted in Microsoft Excel, making use of the tabulated biokinetic data and dose coefficients from the software OIR Data Viewer (ICRP 2022). Random numbers were generated in Visual Basic for Applications. The results are presented as the quotient of the effective dose for the standard assumption a) relative to the effective dose for assumptions b), c), and d) with the same measured activity *A* in the whole body, organ or urine sample (unit mSv per mSv). This allows the comparison of different doses which would result from the same monitoring case under the described assumptions. This presentation, with most numbers being greater than 1 instead of the reciprocal values, was chosen for better readability. When in the following a radionuclide is mentioned without specification of material (i.e. absorption type or chemical compound) and/or monitoring interval, the data from Table 1 apply. Since all dose calculations are proportional to the intake, the presented ratios are independent from the intake.

**Table 1:** Effective doses for various exemplary radionuclides, materials, monitoring methods and intervals, calculated as the result for assumption a) (standard assumption) relative to results for assumptions b), c), and d) for the same measured activity (unit mSv per mSv). Results greater than 1 mean an overestimation under the standard assumption when in fact one of the other assumptions applies; results smaller than 1 mean an underestimation. For assumption d), the arithmetic mean of the values equals the values for assumption c)

Radionuclide	Material	Method^a^	Monitoring interval (days)		Assumption b):	Assumption c):	Assumption d): chronic random intake	
			(in brackets: acc. to ISO 20553:2025)		acute intake, random day	chronic equal intake	5th percentile	95th percentile
Iodine								
^131^I	Aerosols F	U	15	(15)	1.31	140	25	340
^131^I	Aerosols F	Th	15	(15)	0.91	1.06	0.90	1.22
^125^I	Aerosols F	U	90	(90)	0.85	13	2.9	32
^125^I	Aerosols F	Th	90	(90)	0.90	1.10	1.02	1.18
Other elements								
^3^H	Gas/vapour V	U	30	(30)	0.80	1.14	0.94	1.33
^14^C	Aerosols M	U	180	(180)	0.99	7.1	2.9	14
^14^C	Gas/vapour F	U	7^b^	(7)	1.10	7.4	1.69	17
^14^C	Gas/vapour F	U	180^b^	(7)	1.00	330	73	840
^14^C	Aerosols BaCO_3_	U	15^c^	(60)	0.97	38	6.8	96
^14^C	Aerosols BaCO_3_	U	60^c^	(60)	1.07	41	7.6	100
^14^C	Aerosols BaCO_3_	U	90^c^	(60)	0.96	36	6.5	92
^32^P	Aerosols M	U	30	(7)	0.85	2.9	1.63	4.9
^35^S	Aerosols M	U	90	(90)	0.96	12	4.3	24
^90^Sr	Aerosols M	U	180^d^	(365)	1.05	2.7	1.95	3.6
^137^Cs	Aerosols M	U	180	(180)	0.97	1.16	1.07	1.28
^137^Cs	Aerosols M	WB	180	(180)	0.96	1.09	1.03	1.17
^223^Ra	Aerosols M	U	7	(---)	0.81	2.3	1.05	4.2
^225^Ac	Aerosols M	U	7	(---)	0.83	1.22	0.82	1.67
^226^Ra	Aerosols M	U	180	(180)	0.96	2.6	1.60	4.2
^238^U	Aerosols M/S	U	180^d^	(365)	1.06	1.55	1.22	2.1
^238^U	Aerosols M/S	L	180^d^	(365)	0.66	0.85	0.83	0.88
^238^U	Aerosols F	U	30	(30)	0.84	4.5	1.47	10
^239^Pu	Aerosols Pu(NO_3_)_4_	U	180^d^	(365)	1.07	1.41	1.24	1.62

^a^ U: urine measurement (24-hour sample), Th: thyroid measurement, WB: whole-body measurements, L: lung measurement

^b^: Monitoring intervals of 7, 90, and 180 days comply with the factor-3 criterion for this radionuclide, material (i.e. absorption type or chemical compound) and monitoring method. An interval of 7 days requires a detection limit of 710 Bq/d, whereas an interval of 180 days requires a detection limit of 12 Bq/d, which is still achievable (ICRP 2016, Table 3.8)

^c^: It is unclear why ISO 20553:2025 states a monitoring interval of 60 days for this radionuclide, material and monitoring method because also an interval of 90 days complies with the factor-3 criterion with approximately the same detectable annual effective dose. Therefore, the calculation was conducted for intervals of 60 days, 90 days and a short interval of 15 days

^d^: There are international recommendations like those in Etherington et al. 2018 which demand at least 2 measurements per year. Therefore, calculations were not conducted for monitoring intervals of 365 days

## Results and discussion

The calculated ratios (effective dose for the standard assumption a) relative to those for the other assumptions) are presented in Table 1. Assuming an acute intake on variable days in each monitoring interval (assumption b)) leads to similar effective doses on average for all investigated radionuclides and measurement methods. In contrast, assuming a chronic intake (of equal activity on each day or of randomly fluctuating activity; assumptions c) and d)) leads to strongly different, namely smaller, effective doses for ^131^I, ^125^I and several other radionuclides when measurements of urine are conducted. Thyroid monitoring of ^131^I and ^125^I does not exhibit such a strong influence of the time pattern of the intake. Small differences between chronic and acute intake, corresponding to ratios between 2 and 5, are found for a variety of radionuclides (such as ^14^C aerosols type M, ^32^P, ^226^Ra) whereas there are also ratios close to 1 for some radionuclides (such as ^3^H, ^137^Cs, ^238^U aerosols type M/S (in contrast to type F)).

**Fig. 2:**
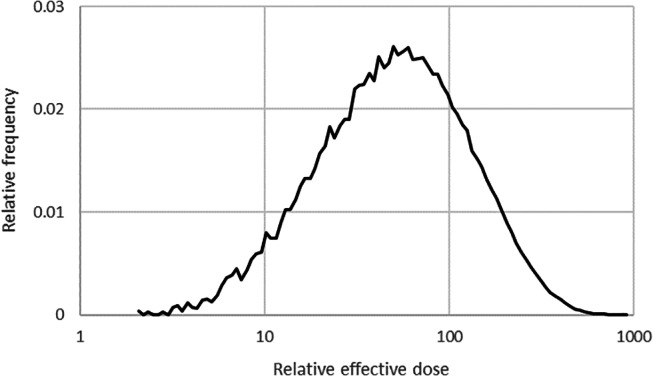
Assumption d) (chronic intake of random activity) for ^131^I, aerosols type F, urine measurements, monitoring interval 15 days: frequency distribution of the effective dose under the standard assumption relative to those randomly calculated under assumption d) (100,000 runs). Arithmetic mean 140, 5th percentile 25, 95th percentile 340

In Fig. 2, the frequency distribution of the dose ratios for assumption d), calculated from the random values of the daily intakes during one monitoring interval, is presented for urine measurements of ^131^I. The values range from slightly greater than 1 (when the intake occurred mainly early during the interval) to almost 1,000 (when the intake occurred mainly late during the interval). The arithmetic mean of this frequency distribution equals the value for assumption c).

**Fig. 3:**
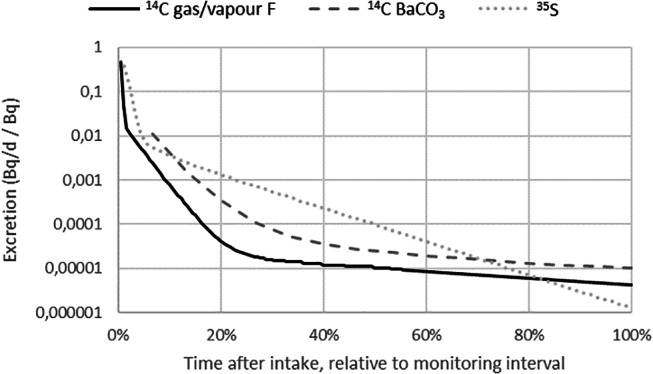
Excretion rates (for urine) of three radionuclides and materials that also feature a strong decrease during the first days after an intake, and which also show a strong influence of the time pattern of the intake on the calculated effective dose as shown in Table 1: ^14^C (gas or vapour type F, monitoring interval 180 d), ^14^C (barium carbonate, BaCO_3_, 15 d) and ^35^S (aerosols type F, 90 d). Values taken from (ICRP 2022)

Of the selected radionuclides, various nuclides beside the iodine isotopes also exhibit a strong dependence of the calculated effective dose on the assumed time pattern of the intake when measured in urine. Among them are ^14^C (gas vapour type F, “unspecified forms”, 180 days, and barium carbonate (BaCO_3_) aerosols also for the short interval of 15 days) and ^35^S (aerosols type M, “barium sulphates, all unspecified forms”, 90 days). All of these radionuclides feature a sharp decrease of the excretion rate during the first days after an intake (Fig. 3), leading to relatively big contributions to the activity in a urine sample from the last days of a monitoring interval, thus leaving only minuscule contributions to the urine activity for the largest earlier part of the interval.

Table 1 indicates that there is a major difference between assumptions b) and c) although both assumptions have in common that intakes are assumed to occur on variable days. This is further explicated in Table 2. There, the calculation of effective doses is conducted for ^131^I for the same monitoring case of a measured activity of 18.2 Bq/d in urine. For assumption b), where an intake occurs on a single variable day in each monitoring interval, most days where an intake is possible lead to an effective dose of approximately 1 mSv, with intakes of approximately 90,000 to 110,000 Bq. Over the course of many monitoring intervals, a mean effective dose of 0.766 mSv will result (in Table 1 represented by the value 1 mSv / 0.766 mSv = 1.31). For assumption c), it was calculated that the comparatively small intake of 647 Bq distributed uniformly over one monitoring interval is sufficient to produce the same activity in the urine sample, because of the large contributions from the daily intakes at the end of the interval; in fact, the intakes during the last two days contribute 97% of the urine activity. This intake yields an effective dose of only 0.00712 mSv, corresponding to its reciprocal value of 140 in Table 1.

**Table 2:** Calculation of the effective dose for ^131^I, aerosols type F, monitoring interval 15 D, under assumptions b) (acute intake on a single random day in each monitoring interval) and c) (chronic intake of the same activity on each day). All of these intakes lead to a measured activity in urine of 18.2 bq/d (for assumption b) each single intake, for assumption c) the sum of the intakes). With this measured activity in urine, an effective dose of 1 mSv is calculated under the standard assumption a) of an acute intake on day 7 (also shown in column “assumption b)” for day 7)

	Assumption b):			Assumption c):	
Day of intake	Intake (Bq)	Effective dose (mSv)		Daily intake (Bq)	Contribution to urine activity (Bq/d)
0	107,000	1.18		43.2	0.00734
1	101,000	1.11		43.2	0.00777
2	101,000	1.11		43.2	0.00777
3	95,700	1.05		43.2	0.00820
4	90,900	1.00		43.2	0.00863
5	90,900	1.00		43.2	0.00863
6	90,900	1.00		43.2	0.00863
7	90,900	1.00		43.2	0.00863
8	95,700	1.05		43.2	0.00820
9	95,700	1.05		43.2	0.00820
10	67,300	0.741		43.2	0.0117
11	15,200	0.167		43.2	0.0518
12	2,040	0.0225		43.2	0.384
13	264	0.00290		43.2	2.98
14	53.5	0.000588		43.2	14.7
			Sum:	647	18.2
	Mean:	0.766	Effective dose:	0.00712	
	Reciprocal: (assumption a) relative to b))	1.31	Reciprocal: (assumption a) relative to c))	140	

For assumption c) (uniform chronic intake), the calculation is simplified when the value of the biokinetic function (retention or excretion) for chronic intake *m*_chronic_(Δ*T*) over an interval of Δ*T* is introduced according to Eq. 6. This definition assumes a constant intake at one point of time per day. A more precise definition, assuming a continuous chronic intake over the entire period of time, can take into account the integral of the values of the biokinetic function over time; however, such biokinetic values must be calculated by means of biokinetic models.

## Conclusions

Whereas a dose calculation at the midpoint of a monitoring interval (which is the standard assumption) is meant to average the calculated doses over the course of several intervals (when acute intakes might occur in some intervals earlier and in some later), it was shown here that this concept can fail severely when intakes occur repeatedly during a monitoring interval or chronically. For radionuclides and monitoring intervals for which the excretion decreases strongly during the first days after an intake, the standard assumption can lead to an overestimation of the calculated effective dose of more than an order of magnitude. This is especially the case when an intake occurred chronically with varying daily contributions, which seems to be a reasonable assumption when subsequent measurements of internal monitoring indicate that intakes at the respective workplace occur frequently. Such an overestimation can have significant adverse effects on the affected individuals and enterprises such as a ban from further work with radioactive material when the annual or lifetime dose limit is exceeded, an unjustified decorporation therapy, psychological strain, or lawsuits against the enterprise. An overestimation of this order of magnitude might not fall under the practice often applied in radiation protection to slightly overestimate radiation doses and, by that, to be on the safe side. As it was shown here, such a huge overestimation cannot be prevented by assuming acute intakes on a single different day per monitoring interval but only by assuming a repeated or chronic intake.

Currently the international standards ISO 20553 and ISO 16637 consider the applicability of an internal monitoring method only regarding a possible *underestimation* of the committed effective dose when it is calculated under the standard assumption (the “factor-3 criterion”, Eqs. 3 and 4 in ISO 20553 (ISO 2025), Eq. 4 in ISO 16637 (ISO 2016)). Despite the unreliability of routine urine monitoring of ^131^I and other radionuclides with regard to dose calculations, routine urine monitoring of these radionuclides would be judged as applicable according to these standards because of the lack of a criterion regarding a possible *overestimation* of the committed effective dose. Consequently, it is recommended that in these standards an additional requirement should be introduced. This requirement should compare the result of the standard dose calculation for an acute intake with that of a dose calculation for chronic intake in its most distinctive form; for the sake of simplicity, constant daily intakes could be hypothetically assumed. This requirement could be expressed as follows (Eq. 9):

9 $$\:\frac{{m}_{\text{chronic}}\left({\Delta\:}T\right)/{\Delta\:}T}{m\left({\Delta\:}T/2\right)}\le\:R$$

where, for example, *R* = 3 or any other reasonable ratio and *m*_chronic_(Δ*T*) is the value of a biokinetic function for chronic intake with constant daily intakes as defined in Eq. 6.

This ratio equals the values in Table 1, column “Assumption c)”.

Whereas the bioassay of urine samples is still a widespread method for the routine internal monitoring of ^131^I, it is recommended here that these measurements are replaced by direct measurements of the activity in the thyroid. The required detection limit for ^131^I, type F, of 190 Bq with a monitoring interval of 15 days would allow detection of an annual effective dose of 1 mSv. An activity of 190 Bq in the thyroid yields a net dose rate of about 5 nSv/h (at 5 cm distance; dose-rate constant from (Delacroix et al. 2002), which is too small to be detected by simple dose-rate meters when they are held close to the thyroid. For dose-rate meters specialised for thyroid measurements, which allow measurement of a net dose-rate of 50 nSv/h within only 10 s, the detection might still be very challenging (Meisenberg and Gerstmann 2017). It is noted, however, that such small activities can reliably be detected with measurement devices that can frequently be found at nuclear medical departments of hospitals: detection limits of about 50–140 Bq can be reached by gamma cameras in a 10 min measurement, and of about 50 Bq can be achieved by thyroid uptake meters in a 1 min measurement (Hjellström et al. 2024; Rodríguez-Laguna et al. 2010). International assistance in providing hospitals with such devices would improve medical diagnostics and the radiation protection of the staff.

For some of the other affected radionuclides, among them ^14^C and ^35^S as pure beta emitters, direct measurements are not an alternative, as it might be also for ^131^I in some cases due to organisational or financial reasons. In these cases, dose calculations should include a thorough consideration of the possible time pattern of the intake even below the investigation level (which is at 30% of the annual dose limit according to ISO 20553 (ISO 2025), taking into account the possibility of repeated or varying chronic intakes, at least when the measurements indicate - after several monitoring intervals - frequent intakes. As it was shown for ^14^C, gas/vapour type F, also the choice of a shorter monitoring interval can mitigate the influence of the time pattern of the intake on the dose, even when longer intervals might still comply with the existing criteria for the applicability of monitoring programmes. However, a surveillance whether an intake has occurred at all can still be conducted with urine measurements in any case provided that the required detection limit is achieved and that the previous caveats regarding a dose calculation are considered.

## Data Availability

No datasets were generated or analysed during the current study.
